# Laboratory evaluation of the Greiner G450 discrete analyser

**DOI:** 10.1155/S1463924687000385

**Published:** 1987

**Authors:** F. Blonde-Cynober, A. Escuret, G. Morgant

**Affiliations:** 1 Laboratoire de Biochimie A – Dr J. Giboudeau – Hôpital Saint Atoine 184 rue du Fg Saint Antoine Paris Cedex 12 F75571 France; 2 Service de Mathématiques Université Paris XI Chatenay-Malabry France


Journal of Automatic Chemistry/*Journal of Clinical Laboratory Automation*, Vol. 9, No. 4 (October–December 1987), pp. 194–197

### Laboratory evaluation of the Greiner G450 discrete analyser

**F. Blonde-Cynober, A. Escuret**

*Laboratoire de Biochimie A – Dr J. Giboudeau – Hôpital Saint Antoine 184, rue du Fg Saint Antoine, F75571 Paris Cedex 12, France*

**and G. Morgant**

*Service de Mathématiques, Université Paris XI, Chatenay-Malabry, France*

*A report of an evaluation of the Greiner G450 selective multichannel analyser is presented. Thirty different single-reagent tests can be carried out in one run at a rate between 200 and 400 tests/h. A double beam photometer allows kinetic and end-point measurements. A Radiometer FLM3 flame photometer has been included for the determination of electrolytes.*

*The G450 analyser demonstrated excellent precision, linearity, accuracy and no carry-over. Results for 16 different analytes as determined with the Greiner G300, Astra 8 Beckman and Progress Kone correlated well with those obtained with the G450.*

### Introduction

The G450 analyser is a selective multichannel system designed for determining enzyme activities and concentrations of substrates and electrolytes. The performance of the analyser was evaluated for 16 variables and the results were compared with those obtained with other instruments in routine use in the authors' laboratory.

### Materials and methods

*Instrumentation*

The sample and the reagents are dispensed into process tubes which are advanced step-by-step in the incubator thermostatted at +30°C and are submitted to an oscillating mixer. The sample volume can vary from 5 to 100 µl. Reagents (up to 30) are kept in a refrigerated compartment. Up to four different reagents can be used for each determination. The volume distributed can vary from 50 to 500 µl. The process tubes are reusable after washing but must be replaced after 10 000 tests. The reaction mixture is transferred to the reading cell with a thermostatted needle. The G450 is equipped with a double-beam filter photometer (eight wavelengths from 334 to 650 nm). The light of the mercury lamp is split into two beams and a thermostatted carroussel with eight quartz cuvettes passes through both light beams. A rotating filter wheel allows the analyser to monitor seven readings at 1 s intervals for each beam and wavelength. With this procedure, kinetic measurements can be performed with polychromatic readings.

The G450 has been integrated with the authors' laboratory computer system – MicroMega 32[R] Alcatel Thomson [1].

The speed of the analyser varies from 200 to 400 tests/h, depending on whether a sample (or reagent) blank is used. A Radiometer photometer was included for the determination of the electrolytes.

*Methods*

Table 1 summarizes the methods, reagents and volumes used with the G450. The same methods were used with

*Table 1. Methods.*

| Test | Method (and reagents*) | Sample (µl) | Total (µl) |
|---|---|---|---|
| Calcium | O. cresolphtalein complex (G) | 20 | 1240 |
| Phosphate | Ammonium molybdate, UV - end point (G) | 20 | 510 |
| Creatinine | Jaffe (G) | 120 | 660 |
| Uric acid | Uricase/4 aminophenazone (G) | 20 | 570 |
| Glucose | Glucose oxidase (G) | 5 | 455 |
| Urea | Urease/Berthelot (G) | 10 | 1105 |
| Iron | Bathophenanthroline (G) | 200 | 500 |
| Total proteins | Biuret (G) | 10 | 560 |
| AST/ALT | IFCC at 366 nm (B) | 50 | 650 |
| ALP | IFCC at 405 nm (G) | 10 | 510 |
| CK | SFBC at 366/436 nm (M) | 40 | 590 |
| LD | [1]DGKC at 366/436 nm (G) | 20 | 670 |
| GGT | IFCC at 405/546 nm (M) | 40 | 500 |
| Na/K | Flame photometer (G) | 20 | 3900 |

* Reagents sources are as follows.

B: bioMérieux.

G: Greiner Instruments AG.

M: Merck, 6100 Darmstadt, FR Germany.

SFBC: Société Française de Biologie Clinique.

[1]: at 30°C.

the reference instruments, except for urinary urea (diacetyl monoxime), CK (IFCC, Baker), LD (Scandinavian recommendations, Baker), ALP (IFCC, Baker), creatinine (Jaffe kinetic, Beckman) and plasma Na/K (specific electrode, Beckman).

*Calibration*

For the determination of enzymatic activities, factors were selected from those recommended in the corresponding non-automatized method. For other analytes, standard curves were established by use of aqueous standards. The following controls were used: Zymotrol EX (bioMerieux), stabilized sera (D1, D2, D3, Decisions, Beckman).

### Results

*Photometer performance*

The linearity of the photometer was tested with an absorbing solution at 405 nm [p.nitrophenol (PNP), 2·16 mmol/l in NaOH 10 mmol/l]. Each measurement was made in duplicate. The absorbance varied linearly with concentrations between 0 and 4 absorbance units (A). The coefficient of variation (CV) was less than 1.00% between 0 and 2·5 A and 1·00 to 5·13% for the upper absorbances.

The linearity of the photometer was tested for all eight wavelengths by Degiampetro *et al.* [2]: the absorbance varied linearly between 0 and 2 A with a CV of less than 0·4% for all wavelengths.

*Accuracy of sample pipetting*

This assay was performed by measuring the absorbance at 405 nm after sampling various volumes of PNP (0·1 mmol/l in NaOH 10 mmol/l) : 5 to 100 µl. The volume of diluant was also varied (distilled water: 50 to 200 µl). Each measurement was performed 10 times. The CVs were less than 1·6% and the percentage of the theoretical volume was between 94 and 102·5%.

*Accuracy of reagent dispenser*

This assay was performed by measuring the absorbance at 405 nm after dispensing various volumes of PNP (0·216 mmol/l in NaOH 10 mmol/l): 50 to 500 µl. Each measurement was performed five times. The CVs were less than 1% and the percentage of theoretical volume was between 97 and 99%.

*Within-run and between-run accuracy*

Within-run accuracy was tested using three pools of serum (or plasma for glucose analysis) and urine from patients at the hospital: L (low), M (medium) and H (high). Thirty consecutive measurements were made for each pool and each parameter. Results (means, standard deviations [SD] and CVs) are given in tables 2(*a*) and 2(*b*). Between-run precision was tested using the above pools in 30 aliquots, for the determination of analyte concentrations, stored at −80°C. For the determination of enzyme activities 20 aliquots, were used with the exception of ALT and LD for which a decrease was observed during storage at −80°C. Stabilized solutions (Decisions) were used for these parameters. On each consecutive day, one aliquot was thawed and analysed for each parameter at the three levels (L, M, H). The results are shown in tables 3(*a*) and 3(*b*).

*Table 2(a). Within-run precision: determination of analyte concentrations (N = 30).*

| Parameter | Pool | Mean | SD | CV% |
|---|---|---|---|---|
| Calcium | L | 2·00 | 0·02 | 1·00 |
| (mmol/l) | M | 2·33 | 0·03 | 1·13 |
| | H | 2·87 | 0·08 | 2·79 |
| Phosphate | L | 0·38 | 0·01 | 2·63 |
| (mmol/l) | M | 0·92 | 0·02 | 2·17 |
| | H | 2·10 | 0·02 | 0·95 |
| Creatinine | L | 51·98 | 0·87 | 1·67 |
| (µmol/l) | M | 85·49 | 1·28 | 1·50 |
| | H | 279·43 | 1·38 | 0·49 |
| Urea | L | 3·60 | 0·07 | 1·89 |
| (mmol/l) | M | 6·03 | 0·06 | 1·06 |
| | H | 23·17 | 0·32 | 1·39 |
| Total protein | L | 48·97 | 0·38 | 0·77 |
| (g/l) | M | 61·24 | 0·50 | 0·82 |
| | H | 80·86 | 0·58 | 0·72 |
| Glucose | L | 3·59 | 0·04 | 1·25 |
| (mmol/l) | M | 4·89 | 0·04 | 0·82 |
| | H | 13·71 | 0·13 | 0·95 |
| Iron | L | 6·29 | 0·13 | 2·07 |
| (µmol/l) | M | 14·34 | 0·12 | 0·83 |
| | H | 34·07 | 0·22 | 0·64 |
| Uric acid | L | 132·57 | 2·65 | 1·00 |
| (µmol/l) | M | 242·20 | 1·92 | 0·79 |
| | H | 791·27 | 13·70 | 1·73 |
| Sodium | L | 128·23 | 0·68 | 0·53 |
| (mmol/l) | M | 140·63 | 0·72 | 0·51 |
| | H | 147·23 | 0·63 | 0·43 |
| Potassium | L | 3·23 | 0·05 | 1·55 |
| (mmol/l) | M | 4·00 | 0·00 | 0·00 |
| | H | 5·16 | 0·06 | 1·16 |
| *Urines* | | | | |
| Sodium | L | 18·80 | 0·61 | 3·24 |
| (mmol/l) | M | 58·80 | 0·41 | 0·69 |
| | H | 101·13 | 1·01 | 0·99 |
| Potassium | L | 21·00 | 0·00 | 0·00 |
| (mmol/l) | M | 41·17 | 0·38 | 0·92 |
| | H | 80·51 | 1·06 | 1·31 |
| Urea | L | 24·40 | 0·44 | 1·80 |
| (mmol/l) | M | 149·62 | 1·82 | 1·22 |
| | H | 444·14 | 4·89 | 1·10 |

*Carry-over*

*Sample dispenser:* this assay was performed according to the recommendations of the French Society of Clinical

*Table 2(b). Within-run precision: determination of enzyme activities (N = 30).*

| Parameter | Pool | Mean | SD | CV% |
|---|---|---|---|---|
| AST | L | 17·83 | 0·75 | 4·21 |
| (IU/l) | M | 45·87 | 1·77 | 3·86 |
| | H | 410·90 | 7·96 | 1·94 |
| ALT | L | 15·13 | 1·52 | 10·05 |
| (IU/l) | M | 42·77 | 1·73 | 4·04 |
| | H | 173·83 | 3·55 | 2·04 |
| ALP | L | 56·80 | 2·17 | 3·82 |
| (IU/l) | M | 98·40 | 2·11 | 2·14 |
| | H | 246·77 | 3·01 | 1·22 |
| CK | L | 27·66 | 2·58 | 9·32 |
| (IU/l) | M | 83·10 | 2·20 | 2·65 |
| | H | 407·87 | 6·48 | 1·59 |
| LD | L | 200·27 | 3·30 | 1·65 |
| (IU/l) | M | 413·33 | 4·36 | 1·05 |
| | H | 879·57 | 5·70 | 0·64 |
| GGT | L | 11·83 | 0·59 | 4·99 |
| (IU/l) | M | 43·70 | 0·59 | 1·30 |
| | H | 176·70 | 0·71 | 0·40 |

Chemistry [3]. For each parameter, a specimen with a low concentration (L1) and one with a high concentration (H1) were analysed 10 times consecutively. The H2, H3, L2, L3 sequence was then carried out five times. The means and SD were calculated for L1, L2, L3 and H1, H2, H3. There was no significant difference between H1 and H2, H2 and H3, L1 and L2, L2 and L3 (data not shown).

*Transfer dispenser and cuvettes:* this assay was performed according to the recommendations of the French Society of Clinical Chemistry [3]. One ALT test was inserted between every four LD measurements. This procedure was repeated 10 times. No interference was observed in the determination of LD.

*Linearity of methods*

For enzymatic activities, sera with abnormally high activities were diluted in 9 g/l NaCl. For the other determinations, the analytes were dissolved and diluted in distilled water or 9 g/l NaCl (determination of T. Proteins). All measurements were carried out in duplicate. Results are given in table 4.

*Methods comparisons*

100 sera (plasma for glucose) and urine samples were analysed with the G450 and with equipment used routinely: Progress (K) (Kone), G300 (G) (Greiner), Astra 8 (B) (Beckman), IL 143 (1) (Instrumentation Laboratory), AA II (A) (Autoanalyzer Technicon). The parameters of the regression curve ($y = ax + b$) and the CVs are shown in table 5.

### Discussion

Both within-run and between-run precision was good and in agreement with results obtained using other analysers [2 and 4]. Less good results were obtained in the within-run assays for ALT (CV for L pool: 10·05%) and CK (CV for L pool: 9·32%). With regard to the between-run assays we obtained the highest CV in 4 series: iron (CV for L pool: 5·35%), uric acid (CV for L pool: 8·46%), AST (CV for L pool: 7·30%) and ALT (CV for L pool: 7·85%).

These relatively high CVs can be explained by the very low level of analytes present.

Apart from two parameters (CK and LD), the linearity of the measurements was satisfactory far above values generally found in plasma or serum. The lower range of linearity observed would be due to the use of 9 g/l NaCl for the dilutions instead of inactivated serum.

*Table 3(a). Between-run precision: determination of analyte concentrations (N = 30).*

| Parameter | Pool | Mean | SD | CV% |
|---|---|---|---|---|
| Calcium | L | 1·88 | 0·04 | 2·13 |
| (mmol/l) | M | 2·35 | 0·04 | 1·70 |
| | H | 2·67 | 0·05 | 1·87 |
| Phosphate | L | 0·61 | 0·02 | 3·28 |
| (mmol/l) | M | 1·03 | 0·02 | 1·94 |
| | H | 1·79 | 0·05 | 2·79 |
| Creatinine | L | 70·40 | 2·82 | 4·00 |
| (μmol/l) | M | 100·50 | 3·64 | 3·62 |
| | H | 262·57 | 9·62 | 3·66 |
| Urea | L | 3·23 | 0·13 | 4·02 |
| (mmol/l) | M | 6·56 | 0·18 | 2·74 |
| | H | 21·95 | 0·37 | 1·68 |
| Total protein | L | 54·55 | 0·66 | 1·21 |
| (g/l) | M | 61·19 | 1·00 | 1·63 |
| | H | 72·96 | 0·94 | 1·29 |
| Glucose | L | 3·72 | 0·13 | 3·49 |
| (mmol/l) | M | 4·84 | 0·13 | 2·68 |
| | H | 15·32 | 0·36 | 2·35 |
| Iron | L | 5·61 | 0·30 | 5·35 |
| (μmol/l) | M | 16·16 | 0·48 | 2·97 |
| | H | 38·51 | 0·84 | 2·18 |
| Uric acid | L | 110·13 | 9·32 | 8·46 |
| (μmol/l) | M | 252·63 | 10·14 | 4·01 |
| | H | 699·17 | 18·27 | 2·61 |
| Sodium | L | 133·67 | 0·96 | 0·72 |
| (mmol/l) | M | 141·37 | 1·19 | 0·84 |
| | H | 152·13 | 1·17 | 0·77 |
| Potassium | L | 3·06 | 0·06 | 1·96 |
| (mmol/l) | M | 4·19 | 0·06 | 1·43 |
| | H | 5·51 | 0·06 | 1·09 |

*Table 3(b). Between-run precision: determination of enzyme activities (N = 20).*

| Parameter | Pool | Mean | SD | CV% |
|---|---|---|---|---|
| AST | L | 13·70 | 1·00 | 7·30 |
| (IU/l) | M | 45·47 | 1·53 | 3·36 |
| | H | 183·62 | 1·99 | 1·08 |
| ALT | L | 21·92 | 1·72 | 7·85 |
| (IU/l) | M | 37·40 | 1·80 | 4·81 |
| | H | 73·62 | 2·34 | 3·18 |
| ALP | L | 59·45 | 1·80 | 3·03 |
| (IU/l) | M | 93·47 | 2·07 | 2·21 |
| | H | 173·27 | 2·52 | 1·45 |
| CK | L | 21·25 | 0·98 | 4·61 |
| (IU/l) | M | 59.70 | 2·02 | 3·38 |
| | H | 351·35 | 5·02 | 1·43 |
| LD | L | 195·12 | 3·46 | 1·77 |
| (IU/l) | M | 384·42 | 5·79 | 1·51 |
| | H | 499·92 | 9·73 | 1·95 |
| GGT | L | 13·37 | 0·48 | 3·59 |
| (IU/l) | M | 53·15 | 0·61 | 1·15 |
| | H | 139·95 | 1·21 | 0·86 |

*Table 4. Range of linearity.*

| Test | Upper limit | Test | Upper limit |
|---|---|---|---|
| Calcium | 6 mmol/l | AST | 700 IU/l |
| Phosphate | 10 mmol/l | ALT | 600 IU/l |
| Creatinine | 900 μmol/l | ALP | 700 IU/l |
| Urea | 60 mmol/l | CK | 600 IU/l |
| Total protein | 210 g/l | LD | 900 IU/l |
| Glucose | 50 mmol/l | GGT | 900 IU/l |
| Iron | 700 μmol/l | Sodium | 500 mmol/l |
| Uric acid | 1260 μmol/l | Potassium | 500 mmol/l |

The results obtained with the G450 correlated well with data from other routine instruments; the slope of the regression curve was between 0·89 and 1·11. Efficient cleaning of the cuvettes and sample and transfer dispensers minimized carry-over.

Daily start-up requires 20 min for cuvette calibration, reagent preparation and an additional 20 min for zero setting and daily quality control.

The 30 reagent dispensers need to be cleaned regularly—at intervals related to the stability of the reagents.

*Table 5. Method comparison.*

| *Parameter* | | *a* ($y = ax + b$) | *b* | $r$* |
|---|---|---|---|---|
| *Blood* | | | | |
| Calcium | (B), mmol/l | 1·10 | −0·26 | 0·956 |
| | (G), mmol/l | 1·02 | −0·04 | 0·976 |
| Phosphate | (G), mmol/l | 0·99 | −0·01 | 0·987 |
| Creatinine | (G), μmol/l | 1·07 | −9·72 | 0·989 |
| Urea | (B), mmol/l | 1·11 | 6·35 | 0·996 |
| T. Protein | (B), g/l | 0·96 | 0·72 | 0·986 |
| Glucose | (G), mmol/l | 0·98 | 0·08 | 0·998 |
| Iron | (G), μmol/l | 0·99 | 0·24 | 0·993 |
| Uric acid | (G), μmol/l | 1·04 | −19·08 | 0·981 |
| Sodium | (B), mmol/l | 1·03 | −6·33 | 0·985 |
| Potassium | (B), mmol/l | 1·03 | 0·02 | 0·993 |
| AST | (K), IU/l | 0·93 | 1·30 | 0·977 |
| ALT | (K), IU/l | 0·89 | 0·78 | 0·993 |
| ALP | (K), IU/l | 0·90 | 2·24 | 0·995 |
| CK | (K), IU/l | 1·02 | −3·38 | 0·995 |
| LD | (K), IU/l | 0·93 | 3·92 | 0·991 |
| GGT | (K), IU/l | 0·96 | −1·74 | 0·998 |
| *Urine* | | | | |
| Sodium | (I), mmol/l | 1·02 | −1·70 | 0·999 |
| Potassium | (I), mmol/l | 1·09 | −2·40 | 0·996 |
| Urea | (A), mmol/l | 1·01 | −3·16 | 0·994 |

* Coefficients of correlation.
With $x$ = reference equipment; $y$ = G450.
B = Beckman – Astra 8.
G = Greiner – G300.
K = Kone – Progress.
I = Instrumentation Laboratory – IL 143.
A = Autoanalyzer Technicon – AA II.

The following analyser has had the following malfunctions: transfer-dispenser preheater (twice), water-temperature incubator control (once), blockage of the flame photometer drain (once).

Being both a high-rate routine and stat analyser, the G450 is particularly suited to large public health laboratories.

### Acknowledgements

Thanks go to A. Delaitre and M. T. Gaubert for expert technical assistance.

### References

1. Kowalezyk, R., Morgant, G., Baumann, F. Ch. and Giboudeau, J., *Journal of Automatic Chemistry*, **8** (1986), 211.
2. Degiampetro, P., Peheim, E. and Colombo, J. P., *Clinical Chemistry*, **31** (1985), 302.
3. Naudin, C., Vassault, A. and Truchaud, A., *Information Scientifique du Biologiste*, **10** (1984), 373.
4. Cynober, L., Morgant, G., Morelet, L. and Giboudeau, J., *Clinical Chemistry*, **33** (1986), 123.

